# Sibuya probability distribution and numerical evaluation of fractional-order operators

**DOI:** 10.1007/s13540-026-00547-0

**Published:** 2026-06-29

**Authors:** Nikolai Leonenko, Igor Podlubny

**Affiliations:** 1 School of Mathematics, Cardiff University, Cardiff, CF 24 4AG United Kingdom; 2 BERG Faculty, Technical University of Kosice, Nemcovej 3, 04200 Kosice, Slovakia

**Keywords:** Fractional calculus (primary), Sibuya probability distribution, Monte Carlo, Fractional-order operators, Numerical simulation, 26A33 (primary), 65C05, 65D25

## Abstract

In this work we explore the Sibuya probability distribution, which serves as the basis and the main instrument for numerical simulations of Grünwald–Letnikov fractional derivatives by the Monte Carlo method. We provide three methods for simulating the Sibuya distribution. We also introduce the Sibuya-like sieved probability distributions, and apply them to numerical fractional-order differentiation. Additionally, we use the Monte Carlo method for evaluating fractional-order integrals. The developed methods and tools are illustrated by examples of computation. We provide the MATLAB toolboxes for simulation of the Sibuya probability distribution, and for the numerical examples.

## Introduction

A class of discrete time random walks has been recently introduced for numerical solution of fractional-order partial differential equations, including fractional-order subdiffusion equations with time fractional derivatives of order $\alpha \in (0, 1)$ (see [1, 18] and the references therein). It turns out that the main role in Monte Carlo method for subdiffusion is played by the Sibuya distribution. However, some problems require the order of the time fractional derivative to be greater than one, and thus such problems require the Monte Carlo method for fractional differential operators of order α>1. For example, in fractional-order diffusion-wave equation the order is $\alpha \in (1, 2]$. In such case, the signed probability distributions appear, as in [16]. The space-time duality for fractional diffusion [3] means that the Monte Carlo method for orders greater than one can also be applied to spatial fractional-order derivatives – see [3] and references therein.

This paper is a continuation of [15, 16] and is devoted to using signed discrete distributions (or extended, or negative probabilities – the terminology has not been unified yet) in the framework of the Monte Carlo method for numerical evaluation of fractional-order derivatives.

Our framework is close to the work of Zhang, Li, and Kerns (see Corollary 3.1 ($3^{o}$) in [32]), and also to the one studied by Burgin [5]. Burgin studies a static environment, where the state space Ω can be divided in two irreducible parts $\Omega _{+}$ and $\Omega _{-}$. The elements of $\Omega _{-}$ are called anti-events, and they are usually connected to negative objects: considering a negative object is a negative event. The author then states that an example of negative objects is given by antiparticles, the antimatter counterpart of quantum particles. Anti-events are assigned negative probabilities.

Another interesting interpretation is given by Székely [30], who proves that we can encounter a negative probability if we work with a random variable having a signed distribution. In addition, if *X* has a signed distribution, then there exist two random variables *Y* and *Z* having ordinary (not signed) such that X+Y=Z in distribution. Therefore, *X* can be seen as a “difference” of two ordinary random variables *Z* and *Y*.

In quantum physics Wigner [31], Dirac [7], and Feynman [9] allow probabilities (and also energies) to be negative, in order to side-step the uncertainty principle of quantum mechanics [22].

Concepts of virtual events are known also in psychology [4], economics [12], and medicine [27].

The paper is organized as follows.

We start with recalling how the Sibuya distribution is related to fractional-order differentiation, and focus on developing the methods for simulation of the Sibuya distribution for 0<α<1. We derive three different methods and provide their implementation in a free MATLAB toolbox; all three methods are perfectly comparable, and any of them can be used for practical simulation.

Then we focus on fractional-order differentiation of orders higher than one. In this case the Sibuya distribution becomes a signed probability distribution, and we develop a general Monte Carlo method for numerical evaluation of fractional-order derivatives of order higher than one.

After that we turn our attention to fractional-order integration. It appears that fractional-order integrals can also be evaluated by the Monte Carlo method using a continuous probability distribution closely related to the Sibuya distribution.

The results of computations using the Monte Carlo method are compared with the exact fractional-order derivatives and integrals of the corresponding functions. The examples are also provided as a MATLAB toolbox.

## Grünwald–Letnikov fractional derivative

Let us recall that the Grünwald–Letnikov fractional derivative is a non-local operator defined as [23]2.1whenever the limit exists, where

2.2 $$\begin{aligned} A_h^\alpha f(t) = \frac{1}{h^\alpha } \left( \Delta _h^\alpha f \right) (t) \end{aligned}$$

and

2.3 $$\begin{aligned} \left( \Delta _h^\alpha f \right) (t) = \sum _{k=0}^{\infty } (-1)^k {\alpha \atopwithdelims ()k} f(t-kh), \end{aligned}$$

for sufficiently good functions *f* [23, 2].

Here, the generalized binomial coefficients appear, defined as

2.4 $$\begin{aligned} {\alpha \atopwithdelims ()k} = \frac{(\alpha )_k}{k!}, \end{aligned}$$

where

2.5 $$\begin{aligned} (\alpha )_k = \frac{\Gamma (\alpha +1)}{\Gamma (\alpha +1-k)} = \alpha (\alpha -1) \ldots (\alpha - k + 1) \end{aligned}$$

is the Pochhammer’s symbol.

It is known that for every bounded function *f* the semigroup property holds, i.e.

2.6 $$\begin{aligned} \Delta _h^\alpha \Delta _h^\beta f = \Delta _h^{\alpha +\beta } f, \quad (\alpha , \beta > 0), \end{aligned}$$

and the Grünwald–Letnikov derivative in many cases coincides with the Riemann–Liouville, Caputo, Weyl or Marchaud fractional derivatives [2, 8, 15, 23].

In particular, the paper [2] contains a detailed discussion of higher-order Grünwald approximations of fractional-order derivatives and fractional powers of operators – see references there, and also [16].

## Sibuya distribution

Consider the sequence of independent Bernoulli trials, in which the *k*-th trial has probability of success α/k (0<α<1,   k=1,2,…). Let $Y \in \{1, 2, \ldots \}$ be the number of the trial in which the first success occurs. Then

$$ p_1 = \mathbb {P} (Y=1) = \alpha , $$

$$ p_k = \mathbb {P} (Y=k) = (1-\alpha ) (1-\frac{\alpha }{2}) \ldots (1-\frac{\alpha }{k-1}) \frac{\alpha }{k}, \quad k \ge 2, $$

or, in other words, its probability mass function of *Y* is

3.1 $$\begin{aligned} p_k= &   (-1)^{k+1} \frac{\alpha (\alpha -1) \ldots (\alpha - k + 1)}{k!} = \nonumber \\= &   (-1)^{k+1} { \alpha \atopwithdelims ()k}, \qquad k = 1,2, \ldots , \end{aligned}$$

and its cumulative distribution functions is

3.2 $$\begin{aligned} F_k = \sum _{j=1}^{k} p_j= &   1 - (-1)^{k} {\alpha -1 \atopwithdelims ()k } = 1- \frac{1}{k \, B (k, 1-\alpha )} = \nonumber \\= &   1 - \frac{\Gamma (k - \alpha + 1)}{k \, \Gamma (k) \Gamma (1-\alpha )}, \quad k = 1,2, \ldots , \end{aligned}$$

where $B(x,y) = \Gamma (x) \, \Gamma (y) /\Gamma (x+y)$, x>0, y>0, is Euler’s beta function.

The probability generating function is

3.3 $$\begin{aligned} G_Y (s) = \mathbb {E} \, s^{Y} = \sum _{k=1}^{\infty } s^{k} (-1)^{k+1} {\alpha \atopwithdelims ()k} = 1 - (1-s)^\alpha , \quad |s| < 1. \end{aligned}$$

Note that, as $k \longrightarrow \infty$,

$$ p_k \sim \frac{\alpha }{\Gamma (1-\alpha )} \, \frac{1}{k^{1+\alpha }}, $$

and hence

$$ \mathbb {E} Y = \infty , $$

where $\mathbb {E}$ denotes the mathematical expectation.

The Sibuya random variable is an integer-valued random variable with the probability mass function given by (3.1) (see, e.g. [6, 21, 28, 29]). Sibuya distribution can be simulated using at least three methods.

### Method 1

This method has been presented and used in [15]. We introduce $F_j = \sum _{k=1}^{j} p_k$, see (3.2), where $p_k$ are defined by (3.1). Then $0=F_0< F_1< \ldots< F_j < \ldots$, and $p_j = F_j - F_{j-1}$.

If *U* is a random variable uniformly distributed in (0, 1), then

$$ \mathbb {P} (F_{j-1}< U < F_j) = p_j, $$

and hence to generate the Sibuya random variable $Y \in \{ 1, 2, \ldots \}$, we set

$$ Y = k, \quad \text{ if } F_{k-1}< U < F_k. $$.

### Method 2

Using the definition of the Sibuya distribution we can generate the sequence of independent uniformly distributed random variables $U_1$, $U_2$, ..., $U_k$, ..., and for a fixed $\alpha \in (0,1)$ we have the following algorithm.

*Step 1.* Put Y=1, if $U_1<\alpha$; otherwise, go to Step 2.

*Step 2.* Put Y=2, if $U_1 \ge \alpha$, but $U_2 < \frac{\alpha }{2}$; otherwise, go to Step 3.

*Step 3.* Put Y=3, if $U_1 \ge \alpha$, $U_2 \ge \frac{\alpha }{2}$, but $U_3<\frac{\alpha }{3}$; otherwise, go to...

and so forth.

In other words, we put Y=k ($k \in \{ 1, 2, \ldots \}$) if $U_1 \ge \alpha$, $U_2 \ge \frac{\alpha }{2}$, ..., $U_{k-1} \ge \frac{\alpha }{k-1}$, but $U_k< \frac{\alpha }{k}$.

### Method 3

We consider Sibuya distribution, that is a random variable $Y \in \{1, 2, 3, \ldots \}$, such that

$$\begin{aligned} p_k = \mathbb {P} (Y=k) = (-1)^{k+1} \frac{\Gamma (\alpha + 1)}{k! \, \Gamma (\alpha - k + 1)}, \quad k = 1, 2, 3, \ldots \end{aligned}$$

Note that its probability generating function is

3.4 $$\begin{aligned} G_Y (s) = \mathbb {E} s^{Y} = \sum _{k=1}^{\infty } s^k p_k = 1 - (1-s)^\alpha , \quad 0< s < 1. \end{aligned}$$

Let us also consider a random variable $X \in {1, 2, 3, \ldots }$ with geometric distribution

3.5 $$\begin{aligned} \mathbb {P} (X = k) = q^{k-1} p, \quad k = 1, 2, 3, \ldots \end{aligned}$$

and probability generating function

3.6 $$\begin{aligned} G_X (s) = \mathbb {E} s^X = p s \sum _{k=1}^{\infty } (qs)^{k-1} = \frac{ps}{1- qs}, \quad 0< s < 1/q. \end{aligned}$$

Also we consider the beta distribution $B (\alpha , 1-\alpha )$ with density

3.7 $$\begin{aligned} f_{\alpha } (x) = \left\{ \begin{array}{ll} \frac{x^{\alpha -1}(1-x)^{-\alpha }}{ \Gamma (\alpha ) \, \Gamma (1-\alpha ) } , &  \quad x \in (0, 1) \\ 0, &  \quad x \notin (0, 1) \end{array} \right. \end{aligned}$$

Note that for any fixed $\alpha \in (0, 1)$ we have $\lim _{x \rightarrow 0+} f_{\alpha } (x) = \infty$, and $\lim _{x \rightarrow 1-} f_{\alpha } (x) = \infty$. The particular case when $\alpha = \frac{1}{2}$ is known as arcsin distribution with the probability generating function

3.8 $$\begin{aligned} f_{\alpha } (x) = \left\{ \begin{array}{ll} \frac{1}{ \pi \sqrt{x (1-x)} } , &  \quad x \in (0, 1) \\ 0, &  \quad x \notin (0, 1) \end{array} \right. \end{aligned}$$

Its cdf is of the form

3.9 $$\begin{aligned} F(x) = \frac{2}{\pi } \arcsin (\sqrt{x}) = \left\{ \begin{array}{ll} 0, &  \quad x < 0 \\ \frac{\arcsin (2x-1)}{ \pi } , &  \quad x \in (0, 1) \\ 1, &  \quad x > 1 \end{array} \right. \end{aligned}$$

Sibuya distribution can be interpreted as a randomly mixed geometric distribution. This has been mentioned for Sibuya distribution for the case of support on { 0, 1, 2, ...} by Sibuya himself [28] and later by Kozubowski and Podgorski [14]. Since in the above papers the full proof is not presented, we present the proof here.

#### Theorem 1

Let *B* has a beta distribution $B(\alpha , 1-\alpha )$. Further, conditionally B=p, which has a geometric distribution (3.5)

$$ \mathbb {P} (Y = k | B = p) = p (1-p)^{k-1}, \quad k = 1, 2, 3, \ldots $$

Then, unconditionally, *Y* has a Sibuya distribution with parameter $\alpha \in (0, 1)$.

#### Proof

Using a conditioning argument, we have from (3.4), (3.5), (3.6), and (3.7):

$$\begin{aligned} \mathbb {E} s^Y= &   \int _{0}^{1} \mathbb {E} (s^Y | B = x) f_{\alpha }(x)\, dx = \int _{0}^{1} \sum _{k=1}^{\infty } s^k x (1-x)^{k-1} \frac{x^{\alpha -1} (1-x)^{-\alpha } }{\Gamma (\alpha ) \Gamma (1 - \alpha )} = \\= &   \frac{1}{\Gamma (\alpha ) \Gamma (1 - \alpha )} \sum _{k=1}^{\infty } s^k \int _{0}^{1} x^{\alpha + 1 - 1} (1 - x)^{k - \alpha - 1} dx = \\= &   \frac{1}{\Gamma (\alpha ) \Gamma (1 - \alpha )} \sum _{k=1}^{\infty } s^k B(\alpha + 1, k-\alpha ) =\\= &   \frac{1}{\Gamma (\alpha ) \Gamma (1 - \alpha )} \sum _{k=1}^{\infty } s^k \frac{\Gamma (\alpha + 1) \Gamma (k-\alpha )}{\Gamma (k+1)} =\\= &   \frac{1}{\Gamma (\alpha ) \Gamma (1 - \alpha )} \sum _{k=1}^{\infty } s^k \frac{ \alpha \Gamma (\alpha ) \Gamma (1-\alpha ) (1-\alpha ) (2-\alpha ) \cdots (k-1 -\alpha )}{k!} = \\= &   \sum _{k=1}^{\infty } s^k (1-\alpha ) (1-\frac{\alpha }{2}) (1-\frac{\alpha }{3}) \cdots (1 -\frac{\alpha }{k-1}) \frac{\alpha }{k} =\\= &   \sum _{k=1}^{\infty } s^k p_k(\alpha ) = 1 - (1 - s)^\alpha , \quad |s|<1, \end{aligned}$$

since

$$\Gamma (k-\alpha ) = \Gamma (k-1 + (1 - \alpha )) = \Gamma (1-\alpha ) (1-\alpha ) (2-\alpha ) \cdots (k-1 -\alpha ). $$

□

From Theorem 1, we arrive at the following method of simulation of Sibuya distribution.

*Step 1.* For a given $\alpha \in (0, 1)$, simulate the random variable *B* with the beta distribution $B (\alpha , 1-\alpha )$, denoting the resulting value of *B* by p∈(0,1).

*Step 2.* For p∈(0,1) arising from Step 1, simulate geometric random variable *X* with probability mass function (3.5) and support $\{1, 2, 3, \ldots \}$, denoting the resulting integer by $k \in \{1, 2, 3, \ldots \}$.

*Step 3.* From Theorem 1 we obtain that Sibuya random variable Y=k.

Note that for the particular case of $\alpha = \frac{1}{2}$ the simulation formula is more elegant, since in this case we have

$$ B = \sin ^2 \left( \frac{\pi }{2} U \right) , $$

where *U* has uniform distribution on (0, 1).

### Numerical simulation of Sibuya distribution

The absence of tools for the simulation of the Sibuya distribution motivated us to create the MATLAB implementation [24] of all three methods, described in sections 3.1, 3.2, and 3.3.

**Fig. 1:**
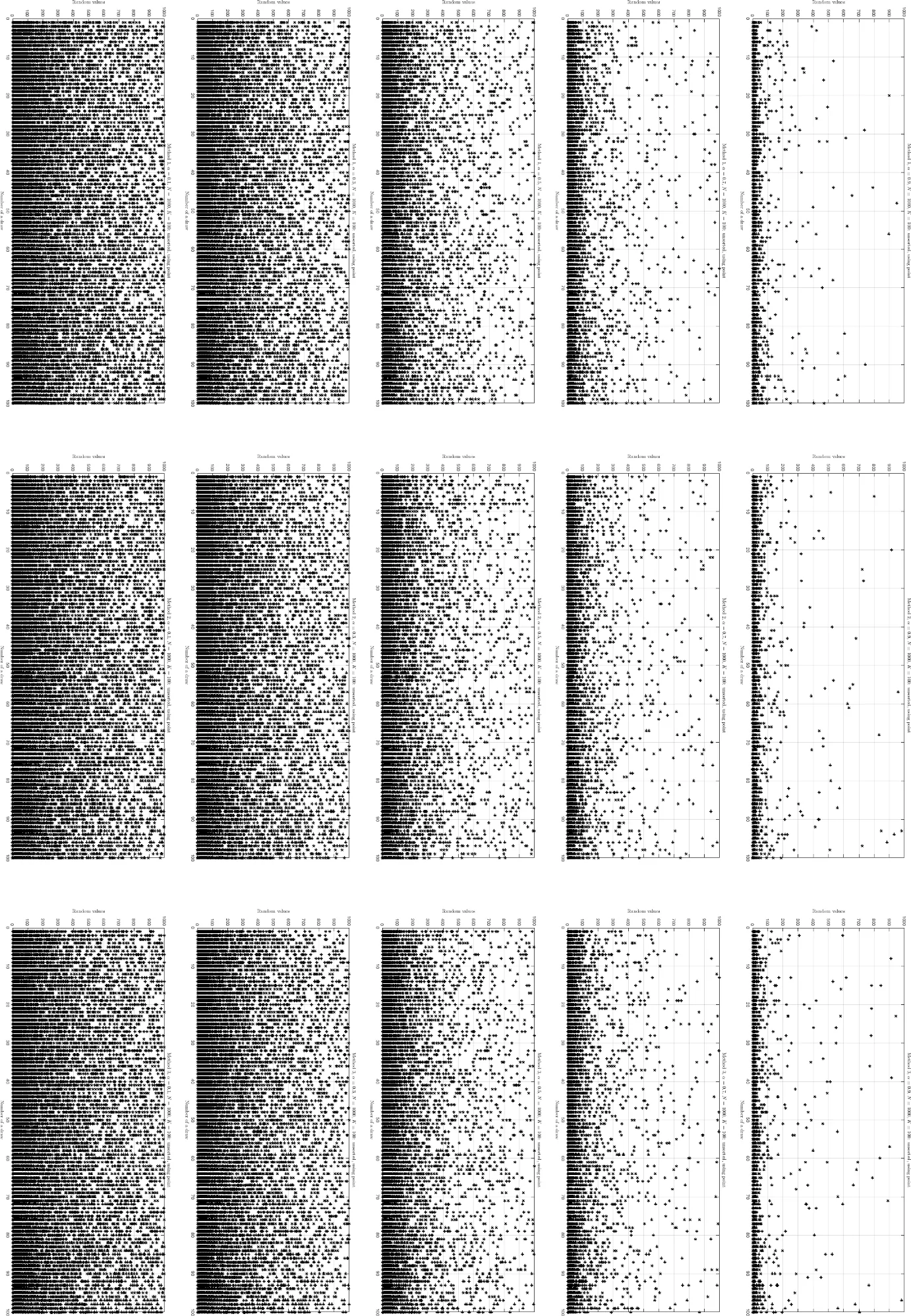
Simulation of the Sibuya distribution by methods 1 (top), 2 (middle), and 3 (bottom) for (left to right) α=0.1, α=0.3, α=0.5, α=0.7, α=0.9, with 100 draws of 1000 numbers each

The examples of the simulations, shown in Fig. 1, provide the visualization of the Sibuya distribution for α=0.1, α=0.3, α=0.5, α=0.7, and α=0.9. All three methods deliver the same character of the distribution of random values, as seen in Fig. 1. Each image there contains one hundred of horizontal sets of 1000 random points (one hundred draws). The density of the random points is higher near the left end (smaller values), and it decreases towards the right end (higher values). For higher values of α the random points concentrate or gravitate, so to say, toward the left end.

**Fig. 2:**
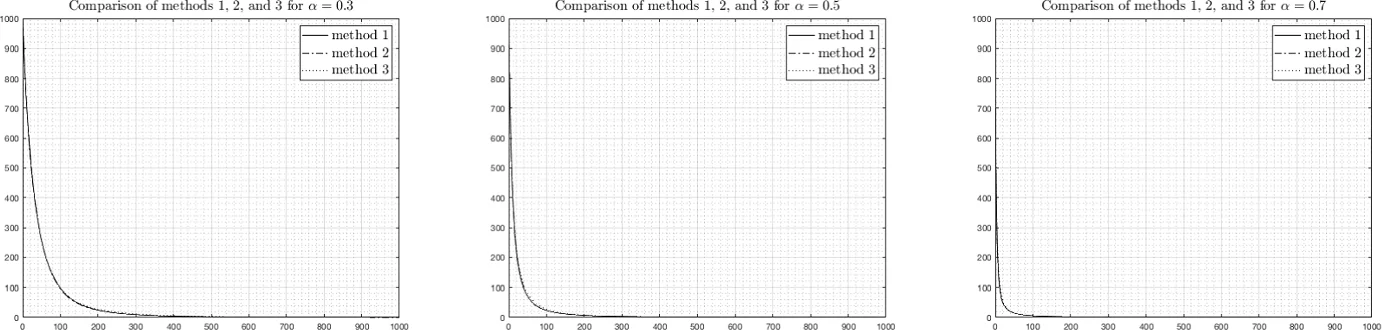
Comparison of the methods 1, 2, and 3 using the sorted averages of the drawn random numbers for α=0.3 (left), α=0.5 (middle), α=0.7 (right)

The three methods are compared using the sorted averages of the the drawn random numbers. To do this, the random numbers of each draw are first sorted, and then average of all draws is computed. The results for α=0.3, α=0.5, α=0.7 are shown in Fig. 2.

The presented three methods of different nature deliver practically identical results, which shows that any of them can be used for simulating the Sibuya distribution. In [17], we have used our methods for simulating the signed probability distributions introduced by Székely [30].

In the current version, provided in the accompanying toolbox, Method 3 is the fastest, and Method 1 is the slowest. The performance of all three methods can change significantly in the case of different implementations.

## Sibuya-like distributions and non-local operators

### The general Monte Carlo procedure

We consider again the non-local operator (2.1), which is known as the Grünwald–Letnikov fractional derivative and could be used (under certain conditions) also for numerical approximation of the Caputo and the Riemann–Liouville fractional derivatives (see, for example, [15, 16, 23]).

We propose a general Monte Carlo scheme for numerical approximations of the operator

4.1 $$\begin{aligned} (\Delta _h^\alpha f) (t) = \sum _{k=0}^{\infty } w_k \, f(t - kh), \quad t> 0, \, \alpha > 0, \end{aligned}$$

where

4.2 $$\begin{aligned} w_k = (-1)^k \frac{ \Gamma (\alpha + 1)}{k! \, \Gamma (\alpha - k + 1)}, \quad k = 0, 1, 2, \ldots \end{aligned}$$

In this section, we address the Monte Carlo approximation schemes for the operator $\Delta _h^\alpha$ in (4.1) for different values of α and for fixed values of *h*, and we confirm numerically that $A_h^\alpha f(t) = (\Delta _h^\alpha f)(t)/h^\alpha$ as h→0 converges to the corresponding fractional-order derivative . Some theoretical results in this direction can be found in [2].

Noting that $w_0 = 1$, and having for |s|≤1 the following binomial series expansion:

$$ (1 - s)^\alpha = \sum _{k=0}^\infty w_k \, s^k, $$

by putting s=1 we have

$$ \sum _{k=0}^\infty w_k = 1 + \sum _{k=1}^\infty w_k = 0, $$

or

4.3 $$\begin{aligned} \sum _{k=1}^\infty w_k = -1, \quad \sum _{k=1}^\infty (-w_k) = 1, \quad \sum _{k=1}^\infty \gamma _k = 1; \end{aligned}$$

but the numbers $\gamma _k = -w_k$ could not be always positive (see [16]).

In order to overcome these difficulties we introduce

$$ I_+ = \{ k \ge 1 : w_k > 0 \}, $$

where we assume that $|I_+| \ge 2$, or $I_+ = \varnothing$, and

$$ W_+ = \sum _{k \in I_+} w_k, $$

then on the discrete set $I_+$, which contains at least two points, we can introduce the discrete distribution

$$ P = \left\{ \frac{w_k}{W_+} , \,\, k \in I_+ \right\} $$

where

$$ p_k > 0, \quad \sum _{ k \in I_+} p_k = 1. $$

Observe that in some cases all $w_k<0$, $k \in \{ 1, 2, \ldots , n, \ldots \} \setminus \{ k^*\}$, that is there exists a single integer $k^*$: $w_{k^*}>0$. Thus, if $w_{k^*}=0$, then the cardinality of the set $I_{+}$ is always greater than or equal two: $| I_{+} | \ge 2$.

Similarly, we consider

$$ I_{-} = \{ k\ge 1: w_k<0 \}, \text{ where } | I_{-} | \ge 2, \text{ or } I_{-} = \varnothing , $$

and we introduce

$$ W_{-} = \sum _{k\in I_{-}}  w_k. $$

Then on the discrete set $I_{-}$ containing at least two points we can introduce the discrete distribution

$$ Q = \left\{ q_k = \frac{w_k}{W_{-}}, \ k \in I_{-} \right\} $$

with

$$ q_k > 0, \quad \sum _{k \in I_{-}}  q_k = 1. $$

Again, it is possible that all $w_k > 0$, $k \in \{ 1, 2, \ldots , n, \ldots \} \setminus \{ k_*\}$, where $k_*$ is a single integer such that: $w_{k_*}< 0$. Thus, if $w_{k_*} = 0$, then $| I_{-} | \ge 2$.

Now we can represent the operator (4.1) as follows:

4.4 $$\begin{aligned} \Delta _h^\alpha f(t)= &   \sum _{k=0}^{\infty } w_k \, f(t - kh) = \nonumber \\= &   f(t) + \sum _{k=1}^{\infty } w_k \, f(t - kh) = \nonumber \\= &   f(t) + W_{+} \sum _{k \in I_{+}} p_k f(t - kh) + w_{k_*} f(t - k_* h) + \nonumber \\  &   + \ W_{-} \sum _{k \in I_{-}} q_k f(t - kh) + w_{k^*} f(t - k^* h) = \nonumber \\= &   f(t) + w_{k_*} f(t - k_* h) + w_{k^*} f(t - k^* h) + \nonumber \\  &   + \ W_{+} \mathbb {E}_P f(t - Y^{+} h) + W_{-} \mathbb {E}_Q f(t - Y^{-} h), \end{aligned}$$

where $w_{k_*} < 0$, $W_{-} < 0$, and assuming that the above expectations exist, $Y^{+}$ is discrete random variable with the support $I_{+}$ and probability mass function $\{ p_k, \, k \in I_{+}\}$, while $Y^{-}$ is discrete random variable with the support $I_{-}$ and probability mass function $\{ q_k, \, k \in I_{-}$}. We always assume that random variables $Y^{+}$ and $Y^{-}$ are independent.

Let $Y_1^{+}$, $Y_2^{+}$, ..., $Y_n^{+}$, ...are independent copies of random variable $Y^{+}$, and $Y_1^{-}$, $Y_2^{-}$, ..., $Y_n^{-}$, ...are independent copies of random variable $Y^{-}$. If $\mathbb {E}_{P} f(t - Y^{+} h) < \infty$, $\mathbb {E}_{Q} f(t - Y^{-} h) < \infty$, then by the strong law of the large number with probability one

4.5 $$\begin{aligned} \frac{1}{N} \sum _{n=1}^{N} f(t - Y_n^{+} h) \longrightarrow \mathbb {E}_{P} f(t - Y^{+} h), \end{aligned}$$

4.6 $$\begin{aligned} \frac{1}{N} \sum _{n=1}^{N} f(t - Y_n^{-} h) \longrightarrow \mathbb {E}_{Q} f(t - Y^{-} h), \end{aligned}$$

as $$N \rightarrow \infty $$.

Consequently, one can show numerically that

4.7 $$\begin{aligned} \left( A_{N,h}^\alpha f \right) (t)= &   \frac{1}{h^\alpha } \Bigl [ f(t) + w_{k_*} f(t - k_* h) + w_{k^*} f(t - k^* h) \Bigr . \nonumber \\  &   + \ W_{+} \frac{1}{N} \sum _{n=1}^{N} f(t - Y_n^{+} h) \nonumber \\  &   \Bigl . + \ W_{-} \frac{1}{N} \sum _{n=1}^{N} f(t - Y_n^{-} h) \Bigr ] \longrightarrow A_h^\alpha f(t), \quad N \rightarrow \infty , \end{aligned}$$

for any α>0, h>0.

The above procedure is the Monte Carlo scheme for (possibly) signed measures $W = \{ w_k, k = 1, 2, \ldots \}$ (see [16]).

We present illustrative examples for the Monte Carlo scheme (4.7) for 0<α<1, 1<α<2, 2<α<3, 3<α<4, and 4<α<5. In particular, the case of 0<α<1 is important for ultraslow diffusion, 1<α<2 is appears in fractional-order diffusion-wave problems, 2<α<3 is related to fractional-order modeling of viscoelastic rods and plates, while other cases are presented for completeness.

### The case of 0<α<1

**Fig. 3:**
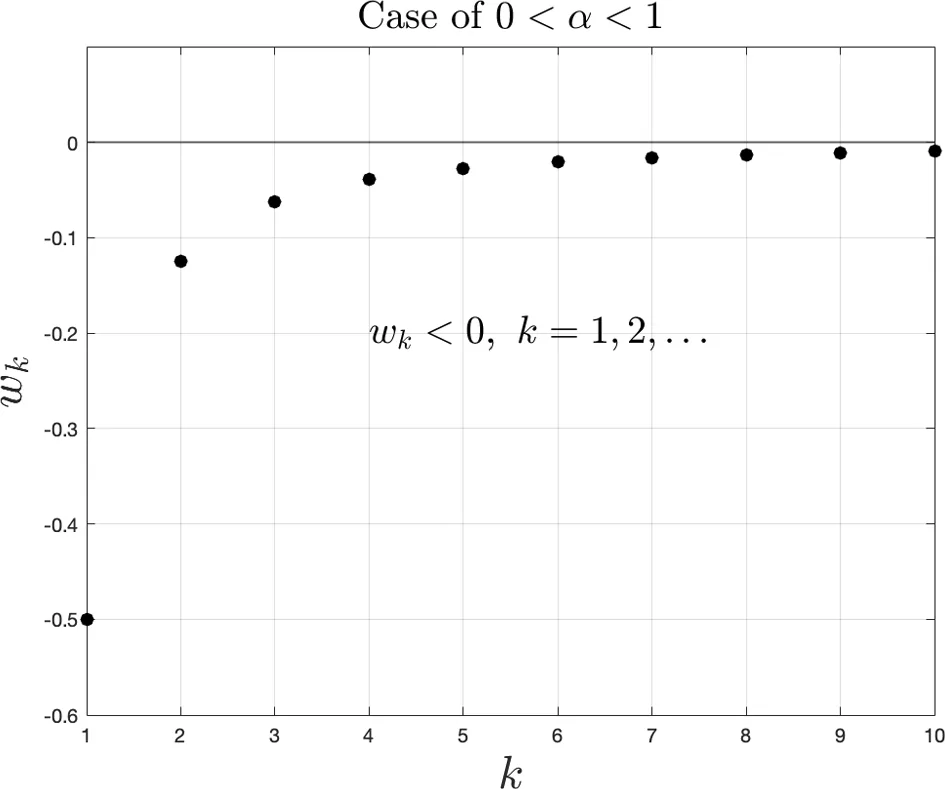
The values of $w_k$, k=1,2,…, in case 0<α<1, with α=0.5

In this case all $w_k < 0$, k=1,2,… (see Fig. 3), thus $I_{+} = \varnothing$, $w_{k^*} = 0$, thus $Y^{+} \equiv 0$, while $I_{-} = \{ k = 1, 2, \ldots : w_k < 0 \} = \{ 1, 2, 3, \ldots , n, \ldots \}$, and $w_{k_*} = 0$. The discrete random variable $Y^{-}$ has the support $\{ 1, 2, \ldots , n, \ldots \}$, and distribution *Q* is Sibuya distribution (see Section 3) with state probabilities

4.8 $$\begin{aligned} q_k = (-1)^{k+1} \frac{\Gamma (\alpha + 1)}{k! \, \Gamma (\alpha - k + 1)}, \quad k = 1, 2, \ldots , \end{aligned}$$

since

$$ W_{-} = \sum _{k \in I_{-}} w_k = 1. $$

The Monte Carlo scheme (4.7) for 0<α<1 takes the form for a fixed *h*:

$$\begin{aligned} (A_{N,h}^\alpha f) (t) = \frac{1}{h^\alpha } \left[ f(t) - \frac{1}{N} \sum _{n=1}^{N} f(t - Y_n^{-} h) \right] \longrightarrow A_h^\alpha f(t) \end{aligned}$$

with probability one as $N \rightarrow \infty$, where $Y_1^{-}$, $Y_2^{-}$, ..., $Y_n^{-}$, ...are independent copies of Sibuya distribution (3.1).

### The case of 1<α<2

**Fig. 4:**
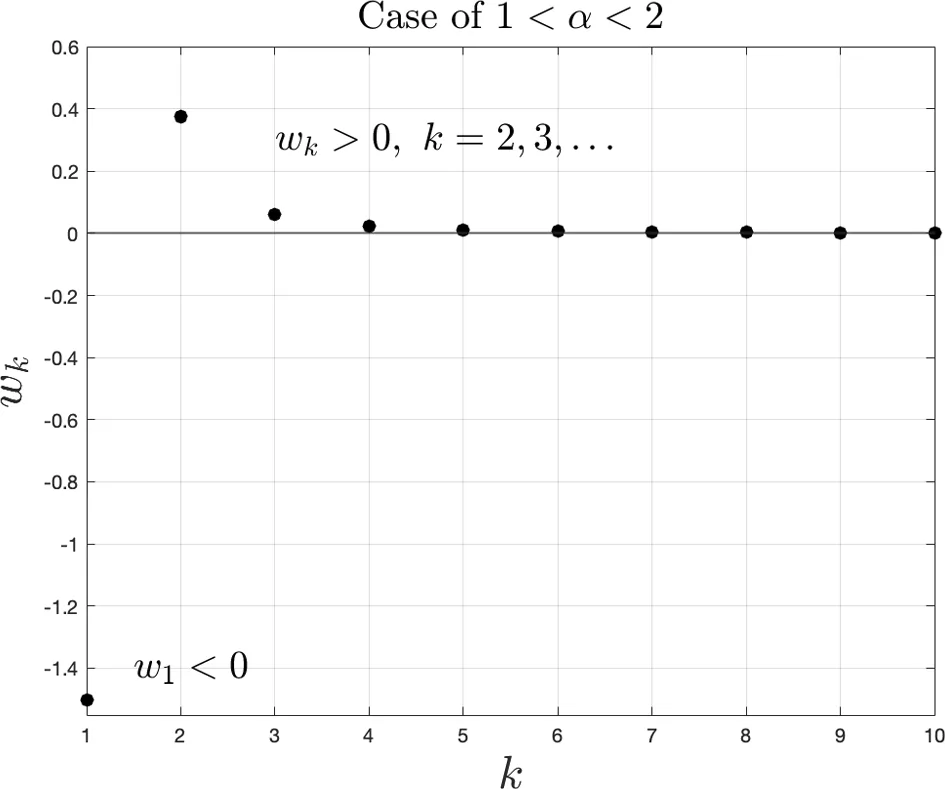
The values of $w_k$, k=1,2,…, in case 1<α<2, with α=1.5

In this case $w_1 = - \alpha < 0$, $w_k > 0$, k=2,3,… (see Fig. 4), thus $I_{+} = \{ k>0: w_k > 0 \} = \{ 2, 3, \ldots \}$, while $k_* = 1$, $w_1 = w_{k_*} = -\alpha$, and $-\alpha + \sum \limits _{k=2}^{\infty } w_k = -1$, hence

$$\begin{aligned} W_{+} = \sum _{k=2}^{\infty } w_k = \alpha - 1 > 0. \end{aligned}$$

Obviously, $$Y^{-} \equiv 0$$, since $$I_{-} = \varnothing $$, $$w_{k^{*}} = 0$$.

Thus, if $Y^{+}$ is the discrete random variable with support $\{ 2, 3, \ldots , n, \ldots \}$, and

4.9 $$\begin{aligned} p_k= &   \mathbb {P} (Y^{+} = k) = \frac{w_k}{W_{+}} = \frac{w_k}{\alpha -1} \nonumber \\= &   \frac{1}{\alpha -1} (-1)^{k} \frac{\Gamma (\alpha + 1)}{k! \, \Gamma (\alpha - k + 1)}, \quad k = 2, 3, \ldots , \end{aligned}$$

and $Y_1^{+}$, $Y_2^{+}$, ..., $Y_n^{+}$, ...are independent copies of random variable $Y^{+}$ given by (4.9), the Monte Carlo procedure takes on the form:

$$\begin{aligned} (A_{N,h}^\alpha f) (t) = \frac{1}{h^\alpha } \Bigl [ f(t) - \alpha f(t-h) + (\alpha -1) \frac{1}{N} \sum _{n=1}^{N} f(t - Y_n^{+} h) \Bigr ] \longrightarrow A_h^\alpha f(t), \end{aligned}$$

with probability one, as $N \rightarrow \infty$.

### The case of 2<α<3

**Fig. 5:**
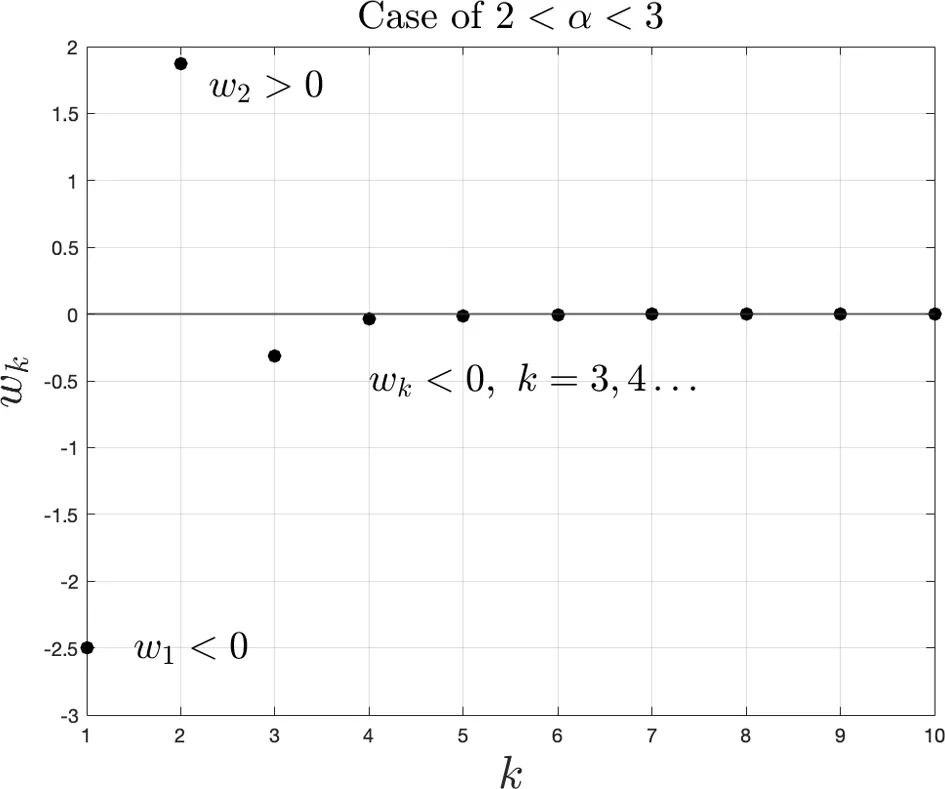
The values of $w_k$, k=1,2,…, in case 2<α<3, with α=2.5

In this case $w_1 = -\alpha < 0$, $w_2 = \frac{\alpha (\alpha -1)}{2} > 0$, $w_k < 0$, k≥3 (see Fig. 5), and $Y^{+} \equiv 0$, since $I_{+} = \varnothing$, but $k^{*}=2$, and $w_{k^{*}}=\frac{\alpha (\alpha -1)}{2}$.

Then $$I_{-} = \{ 1, 3, 4, \ldots , n, \ldots \}$$, and

$$\begin{aligned} -\alpha + \frac{\alpha (\alpha -1)}{2} + \sum _{k=3}^{\infty } w_k = -1, \end{aligned}$$

thus

$$\begin{aligned} W_{-} = -\alpha + \sum _{k=3}^{\infty } w_k = -1 - \frac{\alpha (\alpha -1)}{2} < 0. \end{aligned}$$

We introduce the random variable $Y^{-}$ with support $I_{-} = \{ 1, 3, 4, \ldots , n, \ldots \}$ and state probabilities:

4.10 $$\begin{aligned} q_1 = \frac{-\alpha }{W_{-}} = \frac{2 \alpha }{(2 + \alpha (\alpha -1))} > 0, \end{aligned}$$

4.11 $$\begin{aligned} q_k = \frac{w_k}{W_{-}} = \frac{2 \, (-1)^{k+1} \Gamma (\alpha + 1)}{(2 + \alpha (\alpha -1)) \, k! \, \Gamma (\alpha - k + 1)}, \quad k = 3, 4, \ldots \end{aligned}$$

We call the distribution *Q* of the random variable $Y^{-}$ the *sieved Sibuya-like distribution* for 2<α<3.

We arrive at the following Monte Carlo procedure. If $Y_1^{-}$, $Y_2^{-}$, ..., $Y_n^{-}$, ...are independent copies of the sieved Sibuya-like distribution (4.10)–(4.11), then with probability one as $N \rightarrow \infty$

$$\begin{aligned} (A_{N,h}^\alpha f) (t) =  &   \frac{1}{h^\alpha } \Bigl [ f(t) + \frac{\alpha (\alpha -1)}{2} f(t-2h) - \\  &   - \left( 1 + \frac{\alpha (\alpha -1)}{2} \right) \frac{1}{N} \sum _{n=1}^{N} f(t - Y_n^{-} h) \Bigr ] \rightarrow A_h^{\alpha } f(t) \end{aligned}$$

### The case of 3<α<4

**Fig. 6:**
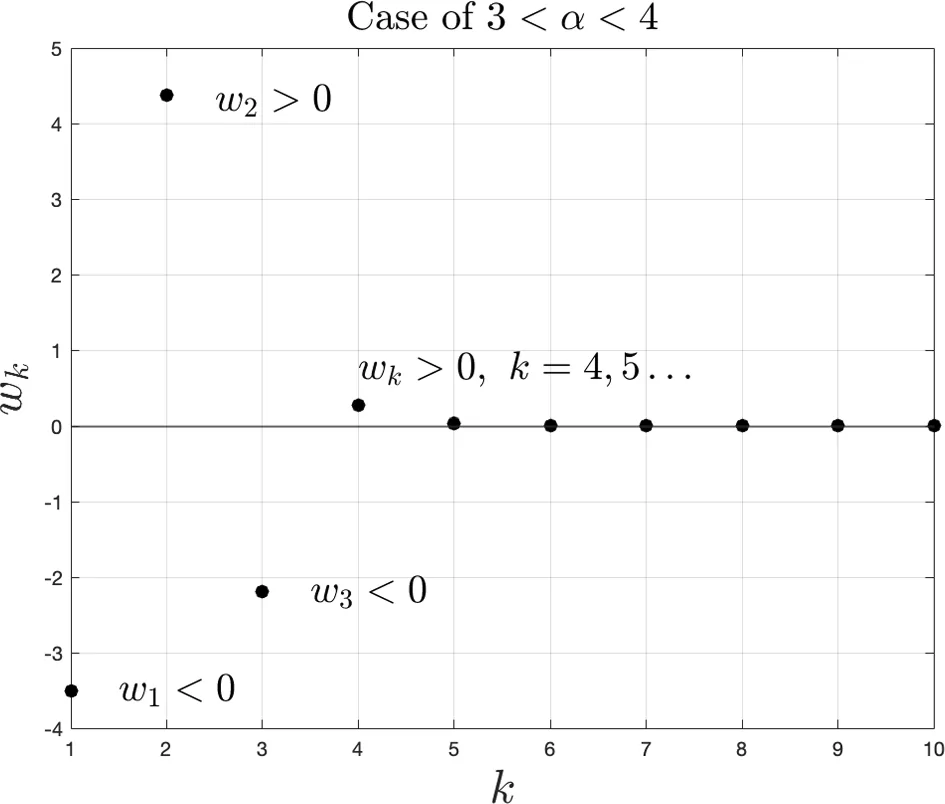
The values of $w_k$, k=1,2,…, in case 3<α<4, with α=3.5

In this case $w_1 = -\alpha < 0$, $w_2 = \frac{1}{2} \frac{\Gamma (\alpha + 1)}{\Gamma (\alpha -1)} > 0$, $w_3 = -\frac{1}{6} \frac{\Gamma (\alpha + 1)}{\Gamma (\alpha -2)} < 0$, $w_k > 0$, k≥4 (see Fig. 6), and

$$\begin{aligned} -\alpha + \frac{1}{2} \frac{\Gamma (\alpha + 1)}{\Gamma (\alpha -1)} -\frac{1}{6} \frac{\Gamma (\alpha + 1)}{\Gamma (\alpha -2)} + \sum _{k=4}^{\infty } w_k = -1, \end{aligned}$$

thus

$$\begin{aligned} W_{+} = w_2 + \sum _{k=4}^{\infty } w_k = \alpha - 1 + \frac{1}{6} \frac{\Gamma (\alpha + 1)}{\Gamma (\alpha -2)} > 0. \end{aligned}$$

Let $Y^{+}$ is the discrete random variable with support $\{ 2, 4, 5, 6, \ldots , n, \ldots \}$ and sieved Sibuya-like distribution *P* for 3<α<4, with state probabilities

$$\begin{aligned} p_2 = \mathbb {P} ( Y^{+} = 2 ) = \frac{\frac{1}{2} \frac{\Gamma (\alpha + 1)}{\Gamma (\alpha -1)}}{\alpha - 1 + \frac{1}{6} \frac{\Gamma (\alpha + 1)}{\Gamma (\alpha -2)} }, \end{aligned}$$

4.12 $$\begin{aligned} p_k = \mathbb {P} ( Y^{+} = k ) = \frac{ (-1)^k \, \Gamma (\alpha +1) \frac{1}{k! \, \Gamma (\alpha - k + 1)} }{\alpha - 1 + \frac{1}{6} \frac{\Gamma (\alpha + 1)}{\Gamma (\alpha -2)} }, \quad k = 4, 5, \ldots \end{aligned}$$

and $Y_1^{+}$, $Y_2^{+}$, ..., $Y_n^{+}$, ...  are independent copies of random variable $Y^{+}$.

The set $I_{-} = \{ k: w_k < 0 \}$ = { 1, 3 }, and $Y^{-}$ is the random variable with support $\{ 1, 3\}$ and state probabilities

4.13 $$\begin{aligned} q_1 = \mathbb {P} (Y^{-} = 1) = \frac{\alpha }{\alpha + \frac{1}{6} \frac{\Gamma (\alpha + 1)}{\Gamma (\alpha -2)} }, \end{aligned}$$

4.14 $$\begin{aligned} q_3 = \mathbb {P} (Y^{-} = 3) = \frac{\frac{1}{6} \frac{\Gamma (\alpha + 1)}{\Gamma (\alpha -2)} }{\alpha + \frac{1}{6} \frac{\Gamma (\alpha + 1)}{\Gamma (\alpha -2)} }, \end{aligned}$$

since $$W_{-} = -\alpha - \frac{1}{6} \frac{\Gamma (\alpha + 1)}{\Gamma (\alpha -2)} < 0$$.

Note that for 3<α<4 we have $w_{k_{*}} = w_{k^{*}}=0$, that is there is no single positive or negative probabilities.

Let $Y_1^{-}$, $Y_2^{-}$, ..., $Y_n^{-}$, ...are independent copies of random variable $Y^{-}$, then as $N \rightarrow \infty$ with probability one we have the following Monte Carlo procedure:

$$\begin{aligned} (A_{N,h}^\alpha f) (t) =  &   \frac{1}{h^\alpha } \left[ f(t) + \left( \alpha -1 + \frac{1}{6} \frac{\Gamma (\alpha + 1)}{\Gamma (\alpha -2)} \right) \frac{1}{N} \sum _{n=1}^{N} f(t - Y^{+} h) \right. \\  &   \hspace{4em} \left. - \left( \alpha + \frac{1}{6} \frac{\Gamma (\alpha + 1)}{\Gamma (\alpha -2)} \right) \frac{1}{N} \sum _{n=1}^{N} f(t - Y^{-} h) \right] \longrightarrow A_h^{\alpha } f(t). \end{aligned}$$

### The case of 4<α<5

**Fig. 7:**
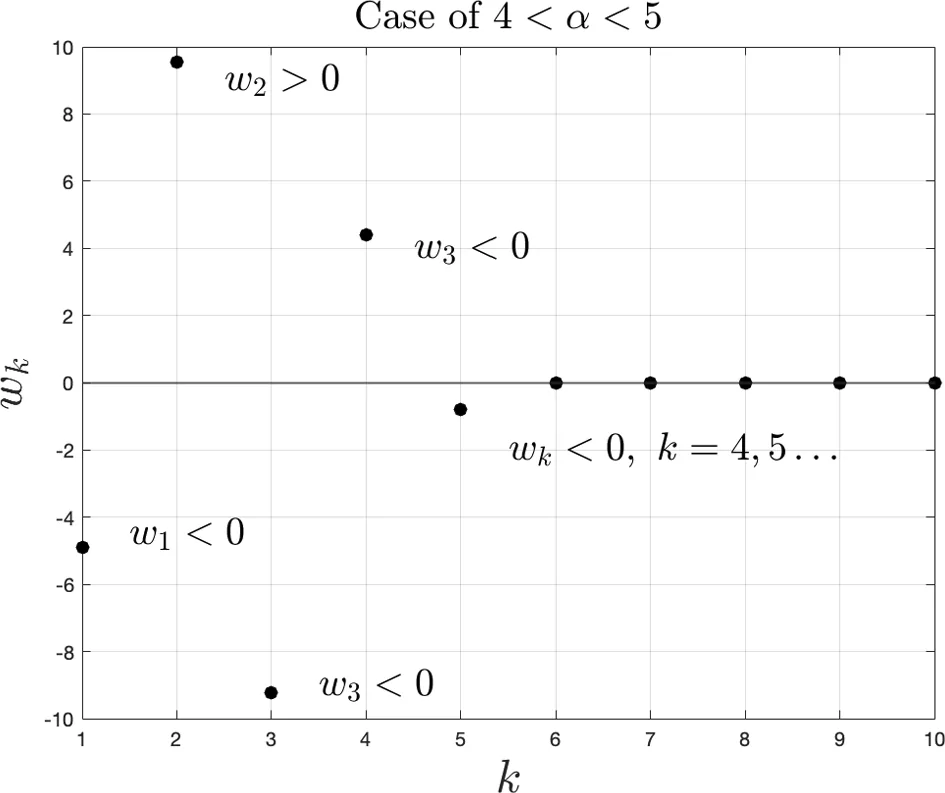
The values of $w_k$, k=1,2,…, in case 4<α<5, with α=4.5

In this case $w_1 = -\alpha < 0$, $w_2 = \frac{1}{2} \frac{\Gamma (\alpha + 1)}{ \Gamma (\alpha -1)} > 0$, $w_3 = -\frac{1}{6} \frac{\Gamma (\alpha + 1)}{\Gamma (\alpha -2)} < 0$, $w_4 = \frac{1}{24} \frac{\Gamma (\alpha + 1)}{\Gamma (\alpha -3)} > 0$, $w_k < 0$, k=5,6,… (see Fig. 7).

Thus, $$I_{+} = \{ 2, 4 \}$$,

$$ W_{+} = \frac{1}{2} \frac{\Gamma (\alpha + 1)}{ \Gamma (\alpha -1)} + \frac{1}{24} \frac{\Gamma (\alpha + 1)}{\Gamma (\alpha -3)} > 0, $$

and random variable $Y^{+}$ has the support $I_{+} = \{ 2, 4 \}$ with probabilities

4.15 $$\begin{aligned} p_2 = \mathbb {P} (Y^{+} = 2) = \frac{\frac{1}{2} \frac{\Gamma (\alpha + 1)}{ \Gamma (\alpha -1)}}{ \frac{1}{2} \frac{\Gamma (\alpha + 1)}{ \Gamma (\alpha -1)} + \frac{1}{24} \frac{\Gamma (\alpha + 1)}{\Gamma (\alpha -3)}}, \end{aligned}$$

4.16 $$\begin{aligned} p_4 = \mathbb {P} (Y^{+} = 4) = \frac{\frac{1}{24} \frac{\Gamma (\alpha + 1)}{\Gamma (\alpha -3)}}{ \frac{1}{2} \frac{\Gamma (\alpha + 1)}{ \Gamma (\alpha -1)} + \frac{1}{24} \frac{\Gamma (\alpha + 1)}{\Gamma (\alpha -3)}}, \end{aligned}$$

The random variable $Y^{-}$ is the sieved Sibuya-like distribution for 4<α<5 with its support

$$ I_{-} = \{ 1, 3, 5, 6, \ldots \}, $$

and

$$ -\alpha + \frac{1}{2} \frac{\Gamma (\alpha + 1)}{ \Gamma (\alpha -1)} -\frac{1}{6} \frac{\Gamma (\alpha + 1)}{\Gamma (\alpha -2)} + \frac{1}{24} \frac{\Gamma (\alpha + 1)}{\Gamma (\alpha -3)} + \sum _{k = 3}^{\infty } w_k = -1, $$

hence

$$\begin{aligned} W_{-} = \sum _{k \in I_{-}} w_k = -1 - \frac{1}{2} \frac{\Gamma (\alpha + 1)}{ \Gamma (\alpha -1)} - \frac{1}{24} \frac{\Gamma (\alpha + 1)}{\Gamma (\alpha -3)} < 0 \end{aligned}$$

and

4.17 $$\begin{aligned} \mathbb {P} (Y^{-} = 1) = \frac{\alpha }{ 1 + \frac{1}{2} \frac{\Gamma (\alpha + 1)}{ \Gamma (\alpha -1)} + \frac{1}{24} \frac{\Gamma (\alpha + 1)}{\Gamma (\alpha -3)}} \end{aligned}$$

4.18 $$\begin{aligned} \mathbb {P} (Y^{-} = 3) = \frac{ \frac{1}{6} \frac{\Gamma (\alpha + 1)}{ \Gamma (\alpha -2)} }{ 1 + \frac{1}{2} \frac{\Gamma (\alpha + 1)}{ \Gamma (\alpha -1)} + \frac{1}{24} \frac{\Gamma (\alpha + 1)}{\Gamma (\alpha -3)}} \end{aligned}$$

4.19 $$\begin{aligned} \mathbb {P} (Y^{-} = k) = \frac{ \frac{ (-1)^{k+1} \Gamma (\alpha + 1)}{k! \, \Gamma (\alpha - k + 1)} }{ 1 + \frac{1}{2} \frac{\Gamma (\alpha + 1)}{ \Gamma (\alpha -1)} + \frac{1}{24} \frac{\Gamma (\alpha + 1)}{\Gamma (\alpha -3)}}, \quad k = 5, 6, \ldots \end{aligned}$$

We arrive at the following Monte Carlo procedure. Let $Y_1^{+}$, $Y_2^{+}$, ..., $Y_n^{+}$, ...are independent copies of random variable $Y^{+}$ given by (4.15)–(4.16), while $Y_1^{-}$, $Y_2^{-}$, ..., $Y_n^{-}$, ...are independent copies of random variable $Y^{-}$ given by (4.17)–(4.19), then with probability one as $N \rightarrow \infty$

$$\begin{aligned} (A_{N,h}^\alpha f) (t) =  &   \frac{1}{h^{\alpha }} \left[ f(t) + \left( \frac{1}{2} \frac{\Gamma (\alpha + 1)}{ \Gamma (\alpha -1)} + \frac{1}{24} \frac{\Gamma (\alpha + 1)}{\Gamma (\alpha -3)} \right) \frac{1}{N} \sum _{n=1}^{N} f( t - Y_n^{+} h) \right. \\  &   \left. - \left( 1 +\frac{1}{2} \frac{\Gamma (\alpha + 1)}{ \Gamma (\alpha -1)} + \frac{1}{24} \frac{\Gamma (\alpha + 1)}{\Gamma (\alpha -3)} \right) \frac{1}{N} \sum _{n=1}^{N} f( t - Y_n^{-} h) \right] \longrightarrow A_h^\alpha f(t). \end{aligned}$$

### General case

The following elegant interpretation and generalization of the above scheme was indicated by one of the four reviewers of this paper; we highly appreciate such an approach.

Recall that

$$\begin{aligned} p_1 = \alpha , \quad p_k = \Bigl (1 - \alpha \Bigr ) \left( \frac{2-\alpha }{2} \right) \left( \frac{3-\alpha }{3} \right) \cdots \left( \frac{k-1-\alpha }{k-1} \right) \left( \frac{\alpha }{k} \right) , \quad k\ge 2, \end{aligned}$$

and hence, for integer $m \in \{ 0, 1, 2, \ldots \}$ and $\alpha \in (m, m+1)$, the coefficients $w_k$ in (4.2), with indices k≥m+1, all have the same sign – they all are positive or they all are negative. Consequently, they can be normalized to define the distribution supported on the shifted set of integers $S_m = \{ m+1, m+2, \ldots \}$. Denote $Y^{(m)} \in S_m$ the generalized Sibuya distribution with the corresponding probabilities

$$\begin{aligned} p_k^{(m)} = \mathbb {P} (Y^{(m)} = k) = (-1)^{k+1} {\alpha \atopwithdelims ()k} / c_m, \quad k \in S_m, \end{aligned}$$

where

$$\begin{aligned} c_m = \sum _{k=m+1}^{\infty } (-1)^{k+1} {\alpha \atopwithdelims ()k} . \end{aligned}$$

Under this formulation, the operator in (4.1) can be written as

$$\begin{aligned} \Delta _h^\alpha f(t)= &   \sum _{k=0}^{m} w_k f(t-kh) \pm c_m \sum _{k=m+1}^{\infty } p_k^{(m)} f(t-kh) = \\= &   \sum _{k=0}^{m} w_k f(t-kh) \pm c_m \mathbb {E} f (t - h \, Y^{(m)}), \end{aligned}$$

where “+” corresponds to odd *m*, and “−” corresponds to even *m*. For m=0, the generalized Sibuya distribution $Y^{(m)}$ reduces to *Y*.

This representation naturally leads to the following Monte Carlo approximation scheme: simulate N≥1 independent copies $Y_1^{(m)}$, $Y_2^{(m)}$, ..., $Y_N^{(m)}$, of the generalized Sibuya distribution, then with probability one as $N \rightarrow \infty$

$$\begin{aligned} \sum _{k=0}^{m} w_k f(t-kh) \pm c_m \frac{1}{N} \sum _{n=1}^{N} f (t - h \, Y_n^{(m)}) \rightarrow \Delta _h^{\alpha } f(t). \end{aligned}$$

Simulation of the generalized Sibuya distributions can be done using, for example, Method 1, with the corresponding modifications. We postpone the numerical implementation of the general method described in this section for two reasons:

1. for the most important case for applications, with $\alpha \in (0, 2]$, the above algorithm reduces to the same as provided in Sections 4.1–4.3;
2. the interpretation of the theory as in Sections 4.1–4.6 could be useful for more general no-local operators – see, for example, the paper [13, formula (3.3)], where a semi-fractional Sibuya distribution is introduced. We plan to discuss this issue elsewhere.

## The Monte Carlo method for numerical fractional integration

Since fractional-order differentiation and integration are closely related and can be considered the unifying generalization of repeated integration and differentiation, we can consider also numerical evaluation of the Riemann-Liouville fractional integrals.

Let us consider the Riemann-Liouville fractional integral of order α:5.1Changing the variable τ=tu allows to write Riemann-Liouville fractional integral (5.1) in the form of the Stieltjes integral:5.2or5.3where the function

5.4 $$\begin{aligned} G(u) = \left\{ \begin{array}{ll} 0, &  u \in (-\infty , 0) \\ 1- (1-u)^{\alpha }, &  u \in [0, 1) \\ 1, &  u \in [1, \infty ) \end{array} \right. \end{aligned}$$

is the cumulative function for the random variable with probability density

5.5 $$\begin{aligned} g(u) = \left\{ \begin{array}{ll} \alpha (1-u)^{\alpha - 1}, &  x \in (0, 1) \\ 0, &  x \not \in (0, 1) \end{array} \right. \end{aligned}$$

The function *G*(*u*) reminds the generating function for the discrete Sibuya distribution used in the previous sections for numerical evaluation of fractional derivatives, and this emphasizes that fractional-order derivatives and fractional-order integrals are, so to say, “growing from the same root”. A close look at the above shows that this distribution is a particular case of the beta-distribution $B(\mu , \alpha )$ for μ=1, and *G*(*u*) is a particular case of the regularized incomplete beta function: $G(u) = I_u(1, \alpha )$.

We observe here a nice duality of the discrete Sibuya distribution and a related continuous probability distribution (in this case, a particular case of the beta distribution). A more general case has been introduced and studied recently by Ohemeng and Kozubowski [19] as discrete-continuous generalized Sibuya dual pair of distributions.

Therefore, we have the following algorithm for the Monte Carlo method for fractional integration.

- **Step 1.** Generate *N* random points $X_i$ using *G*(*u*).
- **Step 2.** Compute
- **Step 3.** Repeat steps 1 and 2 in *K* trials.
- **Step 4.** Compute the mean of those *K* trials, which gives the approximate value of the fractional-order integral (5.1).

## Examples

In the examples provided in this section the results of computations using the Monte Carlo method are compared with the exact fractional-order derivatives and integrals of the corresponding functions. In each figure, the solid line represents the exact fractional-order derivative or integral, the small dots arranged vertically are the results of the repeated trials, the bold dot is the evaluated value (the mean of the trials), and the short horizontal lines correspond to confidence interval for the computed value represented by the bold dot.

In the examples provided below the Mittag-Leffler function with two parameters is used,

$$ E_{\delta , \beta }(z) = \sum _{k=0}^{\infty } \frac{z^k}{\Gamma (\delta k + \beta )}, \quad (\delta> 0, \,\, \beta > 0), $$

for which we have [23, pp. 17–18]:

$$ E_{1, 1}(z) = e^z, \qquad z^2 E_{1,3}(z) = e^z - 1 - z, $$

$$ z E_{2, 2} (-z^2) = \sin (z), \qquad E_{2, 1} (-z^2) = \cos (z). $$

We also have a convenient rule for its fractional-order differentiation and integration [23, p. 310]:The Mittag-Leffler function is computed using the MATLAB function [25], and the examples are included in [26]. One can also use the excellent MATLAB function developed by R. Garrappa [10, 11].

### Example 1: fractional differentiation of order 0<α<1.

6.1 $$\begin{aligned} y(t) = E_{\alpha , 1} (-\lambda t^\alpha ) - 1 \end{aligned}$$

**Fig. 8:**
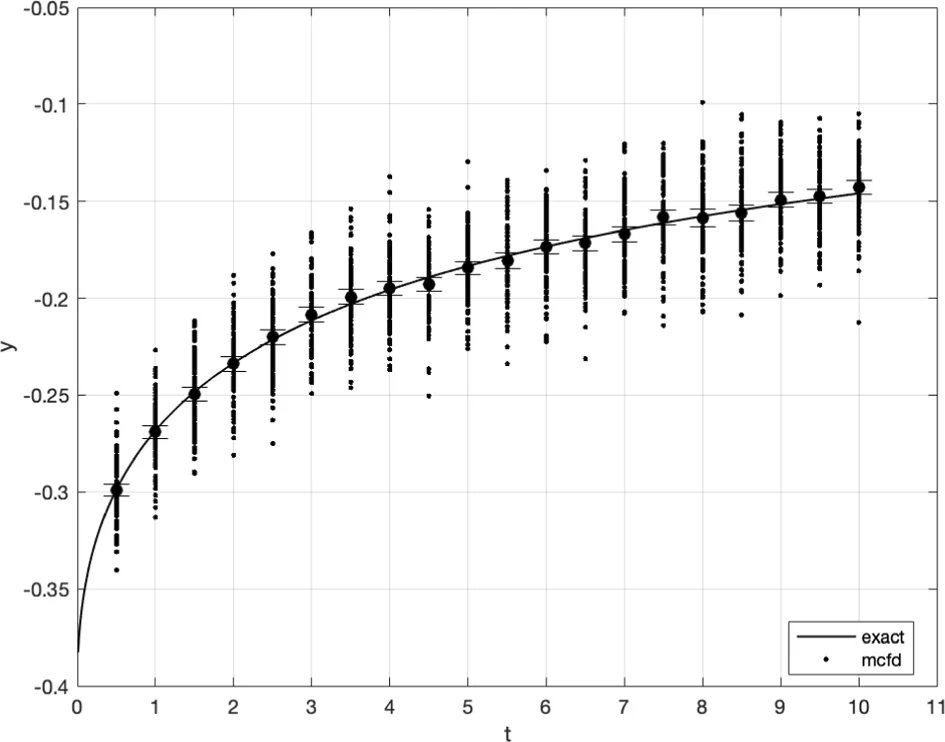
Evaluation of  for $y(t) = E_{\alpha , 1} (-\lambda t^\alpha ) - 1$; α=0.5, λ=0.4

6.2The result of evaluation for α=0.5, λ=0.4 is shown in Fig. 8.

### Example 2: fractional differentiation of order 1<α<2.

6.3 $$\begin{aligned} y (t) = \sin (t) \end{aligned}$$

**Fig. 9:**
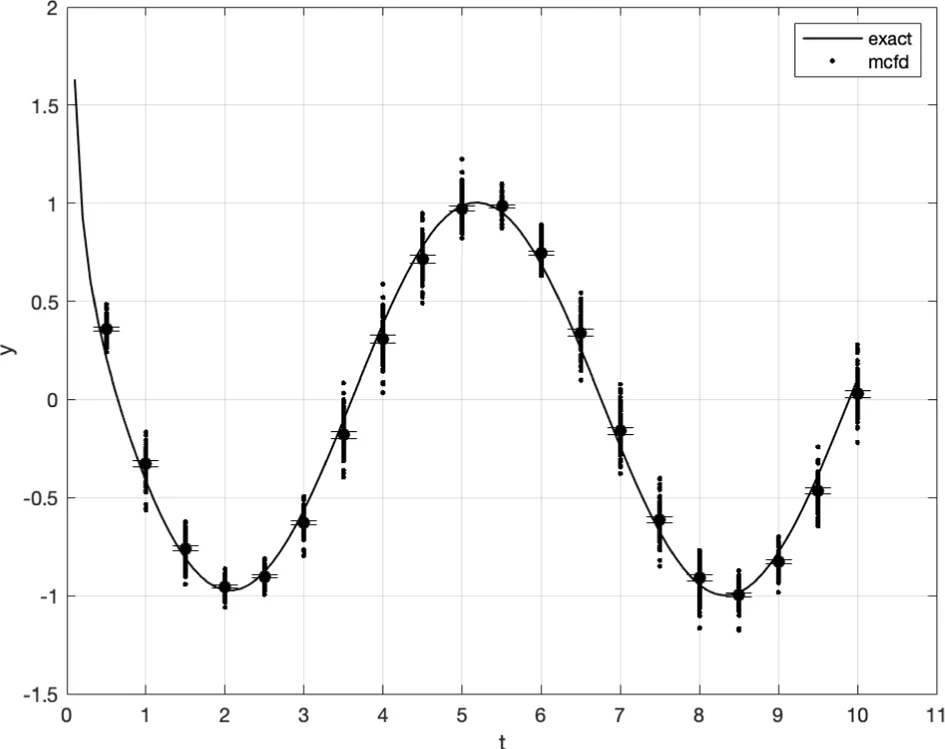
Evaluation of  for y(t)=sin(t); α=1.7

6.4The result of evaluation for α=1.7 is shown in Fig. 9.

### Example 3: fractional differentiation of order 2<α<3.

6.5 $$\begin{aligned} y(t) = e^{-\lambda t} - 1 + \lambda t; \end{aligned}$$

**Fig. 10:**
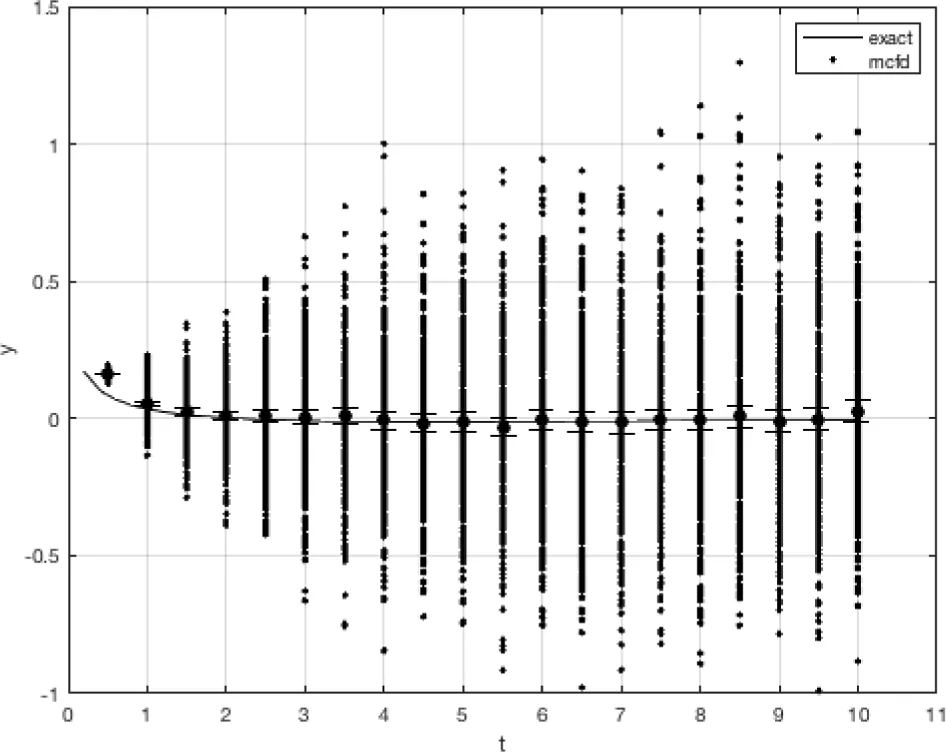
Evaluation of  for $y(t) = e^{-\lambda t} - 1 + \lambda t$; α=2.5, λ=0.4

6.6The result of evaluation for α=2.5 and λ=0.4 is shown in Fig. 10.

### Example 4: fractional integration of order 0<α<1.

6.7 $$\begin{aligned} y(t) = t^{-\alpha } E_{2, 1-\alpha } (-t^2). \end{aligned}$$

**Fig. 11:**
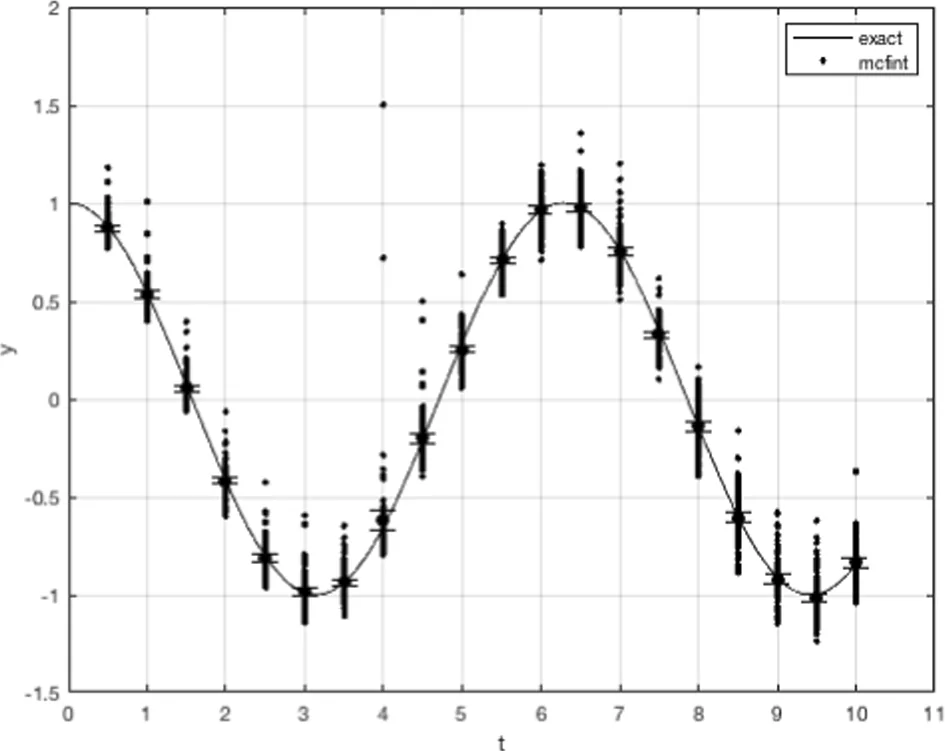
Evaluation of  for $y(t) = t^{-\alpha } E_{2, 1-\alpha } (-t^2)$; α=0.6

6.8The result of evaluation for α=0.6 is shown in Fig. 11.

### Example 5: fractional integration of order 1<α<2.

6.9 $$\begin{aligned} y(t) = t^{1-\alpha } E_{2, 2-\alpha } (-t^2). \end{aligned}$$

**Fig. 12:**
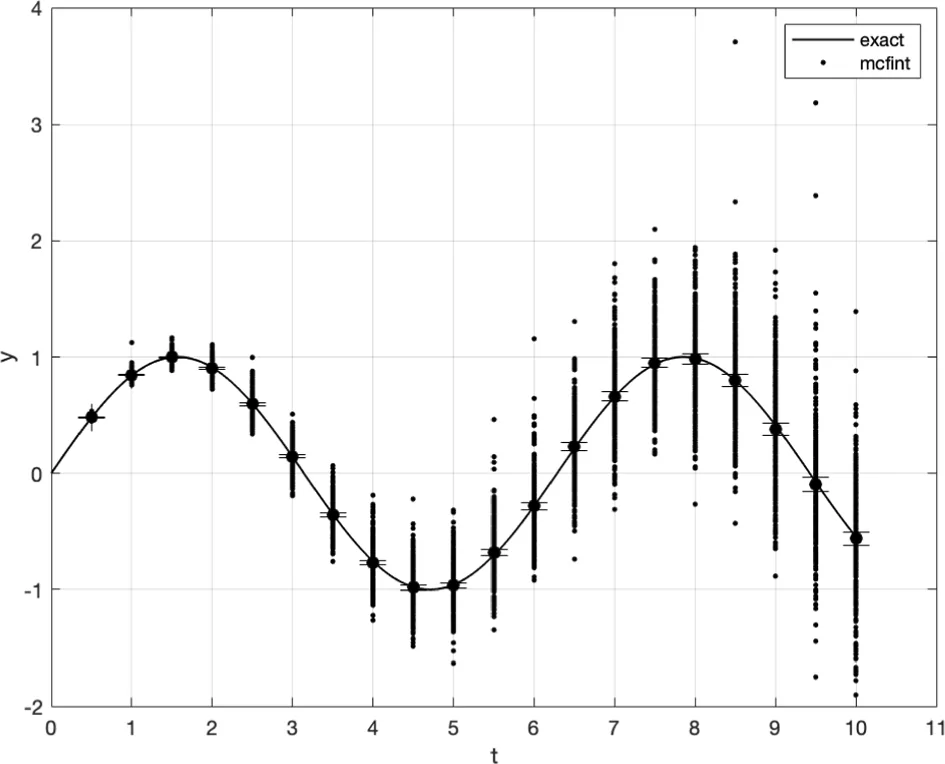
Evaluation of  for $y(t) = t^{1-\alpha } E_{2, 2-\alpha } (-t^2)$; α=1.4

6.10The result of evaluation for α=1.4 is shown in Fig. 12.

### Example 6: fractional integration of order 2<α<3.

6.11 $$\begin{aligned} y(t) = \frac{t^{\nu }}{\Gamma (\nu + 1)} \end{aligned}$$

**Fig. 13:**
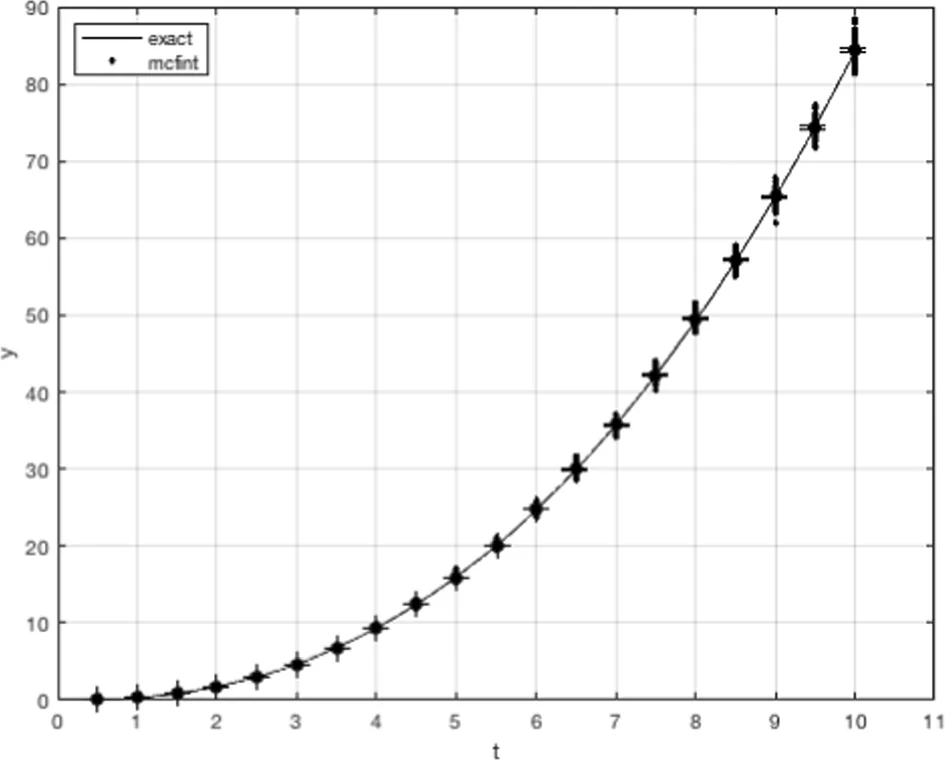
Evaluation of  for $y(t) = \frac{t^{\nu }}{\Gamma (\nu + 1)}$; ν=-0.3, α=2.7

6.12The result of evaluation for ν=-0.3 and α=2.7 is shown in Fig. 13.

## Conclusion

We can conclude that, due to its link to fractional-order differentiation, fractional-order integration, fractional Bernoulli and Poisson processes [20], and related fields, the Sibuya distribution deserves close attention and investigation. Consideration of the case when its parameter is greater than one takes us to the field of signed probability distribution.

For higher orders of fractional differentiation, the simulation of the sieved Sibuya distributions brings computational challenges. Also, the presented Monte Carlo method using signed probabilities can be further enhanced with the help of standard approaches for improving the classical Monte Carlo method. Both aspects require further exploration.

## Data Availability

This article does not report on or use any research data. Data sharing is not applicable to this study.
